# Patient Perceptions of Their Own Data in mHealth Technology–Enabled N-of-1 Trials for Chronic Pain: Qualitative Study

**DOI:** 10.2196/10291

**Published:** 2018-10-11

**Authors:** Robin L Whitney, Deborah H Ward, Maria T Marois, Christopher H Schmid, Ida Sim, Richard L Kravitz

**Affiliations:** 1 Department of Internal Medicine University of California, San Francisco Fresno Medical Education Program University of California, San Francisco Fresno, CA United States; 2 Betty Irene Moore School of Nursing University of California, Davis Sacramento, CA United States; 3 Center for Healthcare Policy and Research University of California, Davis Sacramento, CA United States; 4 Department of Biostatistics School of Public Health Brown University Providence, RI United States; 5 Center for Evidence Synthesis in Health School of Public Health Brown University Providence, RI United States; 6 Division of General Internal Medicine School of Medicine University of California, San Francisco San Francisco, CA United States; 7 Department of Internal Medicine University of California, Davis Sacramento, CA United States

**Keywords:** mHealth, patient-generated health data, self-management, chronic pain, qualitative research, N-of-1 trials, mobile phones

## Abstract

**Background:**

N-of-1 (individual comparison) trials are a promising approach for comparing the effectiveness of 2 or more treatments for individual patients; yet, few studies have qualitatively examined how patients use and make sense of their own patient-generated health data (PGHD) in the context of N-of-1 trials.

**Objective:**

The objective of our study was to explore chronic pain patients’ perceptions about the PGHD they compiled while comparing 2 chronic pain treatments and tracking their symptoms using a smartphone N-of-1 app in collaboration with their clinicians.

**Methods:**

Semistructured interviews were recorded with 33 patients, a consecutive subset of the intervention group in a primary study testing the feasibility and effectiveness of the Trialist N-of-1 app. Interviews were transcribed verbatim, and a descriptive thematic analysis was completed.

**Results:**

Patients were enthusiastic about recording and accessing their own data. They valued sharing data with clinicians but also used their data independently.

**Conclusions:**

N-of-1 trials remain a promising approach to evidence-based decision making. Patients appear to value their roles as trial participants but place as much or more importance on the independent use of trial data as on comparative effectiveness results. Future efforts to design patient-centered N-of-1 trials might consider adaptable designs that maximize patient flexibility and autonomy while preserving a collaborative role with clinicians and researchers.

## Introduction

Evidence-based medicine, as described by Sackett et al (1996), is “the conscientious, explicit, and judicious use of current best evidence in making decisions about the care of individual patients” [1]. At the top of the evidence hierarchy are randomized controlled trials (RCTs) and meta-analyses of RCTs [2]. However, even when such evidence is available, applying it to an individual patient in clinical practice is not always straightforward [3].

Even the highest quality RCTs and meta-analyses can only estimate the average effect of a treatment from a group of trial participants [4]. Because there is inherent heterogeneity in the effects of any treatment (ie, individuals within a population experience differences in the magnitude and direction of treatment and side effects), the average effect estimated in an RCT is not always applicable to an individual patient [4,5]. The uncertainty around applying such evidence may be particularly salient when patients have attributes (eg, complex medical or social circumstances) that differentiate them from an average RCT participant. Several studies have convincingly demonstrated that typical patients in many RCTs (ie, those at the 50^th^ percentile for risk of poor outcomes) differ greatly from and often receive much less benefit than the average patients [3]. Therefore, investigators have sought methods that bring evidence-based practice closer to Sackett’s ideal [1,5].

N-of-1 trials are individualized crossover trials that may help to address this dilemma by comparing the effectiveness of 2 (or more) treatments for an individual patient [6]. N-of-1 trials have the potential to improve clinical decision making in several ways; for example, they can be conducted in a “real world” environment—with clinically complex patients who would likely not meet the stringent inclusion criteria of a traditional RCT—and adapted with individualized protocols that consider the needs and preferences of patients and clinicians [5-7].

Despite many potential advantages, N-of-1 trials are infrequently used in health care [5], partly because of inherent limitations of the method. They are as follows. N-of-1 trials are only applicable to chronic, nonfatal conditions and to treatments with rapid onset and washout. In addition, the appeal of N-of-1 trials has been limited by several external factors including cost, lack of external funding opportunities, and the substantial time investment required from both clinician and patient [5].

Some of these logistical barriers may be overcome with the usage of mHealth technology to collect and synthesize patient-generated health data (PGHD). PGHD is defined as “health-related data created, recorded, or gathered from and by patients (or family members or other caregivers) to help address a health concern” [8]. The explosion of wearables and mHealth technology apps designed to collect and manage PGHD may provide an approach to N-of-1 trial data collection that eases participant burden while providing valuable information that informs decision making and improves patients’ understanding of their own symptoms and treatments.

Patients appear to value and use PGHD obtained from mHealth and wearable technologies. However, few studies have examined patients’ experience with PGHD in the N-of-1 trial setting [7,9,10]. In a study that examined theoretical barriers and facilitators to N-of-1 trial adoption, patients with chronic conditions expressed enthusiasm for the N-of-1 trial concept [7]. In particular, patients appeared to value the idea of having individualized results and felt that participation would have other likely benefits such as more rigorous self-monitoring of symptoms and side effects [7]. However, patients also expressed concerns about potential safety issues and skepticism around feasibility, given the already busy schedules of clinicians and lives of patients [7]. Two previous studies that qualitatively examined perspectives of N-of-1 trial participants reported that patients were largely satisfied with their N-of-1 trial experience and that participation increased self-awareness of their medical condition [9,10]. However, both studies were based in the United Kingdom and neither reported on N-of-1 trials that included an mHealth component.

In this study, we extend prior knowledge by exploring patients’ experiences in monitoring their own symptoms and collaborating with primary care providers to use PGHD resulting from mHealth supported N-of-1 trials for chronic pain management. Our findings shed light on ways in which patients make sense of and use their health data in partnership with clinicians to inform treatment decisions in the N-of-1 trial context. Results of our work may inform targeted patient education, shared decision making, and self-management interventions to improve chronic pain. In addition, our findings have important implications for patient-centered design and implementation of mHealth technology–enabled N-of-1 trials.

## Methods

### Design and Setting

This qualitative study was embedded within a randomized controlled trial, Personalized Research for Monitoring Pain Treatment (PREEMPT), which examined the effectiveness of using a mobile pain software app for conducting single-patient crossover (“N-of-1”) trials among patients with chronic musculoskeletal pain. Members of our team collaborated with the nonprofit mHealth developer Open mHealth to create the app, called Trialist, to promote tracking and summarizing of chronic pain symptoms using a smartphone. The Trialist app allows tracking of chronic pain symptoms over time while also providing graphical summaries of results of N-of-1 comparative trials.

### The Personalized Research for Monitoring Pain Treatment Study

Details of the PREEMPT study design are reported elsewhere [11]. Briefly, a sample of primary care clinicians and their chronic pain patients were recruited. Patients were randomized to the Trialist app intervention or usual care. Both Trialist app intervention and usual care groups had multiple variables measured at baseline and at 3, 6, and 12 months through self-administered questionnaires. Questions assessed pain intensity, pain interference, medication adherence, medication-related shared decision making, pain treatment satisfaction, and general health-related quality of life.

**Figure 1:**
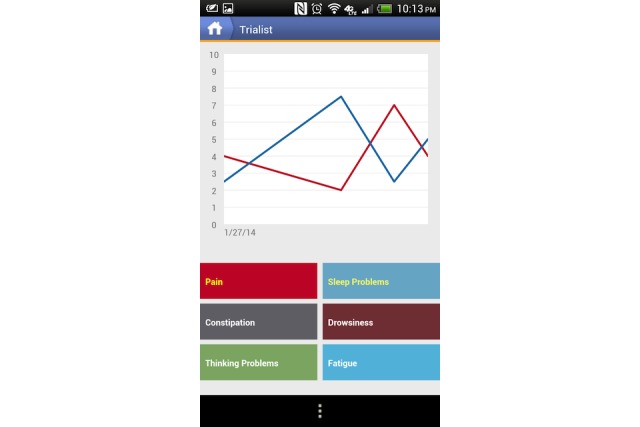
Example of patients’ view within the app.

Intervention patients were provided free access to the app for use on their mobile device (iOS or android). These patients collaborated with their clinicians to create single-patient crossover (N-of-1) trials. In setting up the N-of-1 trial, the patient and clinician would jointly determine treatments to compare length of time for each treatment (1 or 2 weeks) and the number of times the patient would switch between treatment A and B (2, 3, or 4); for example, a patient might use treatment A for 2 weeks and then switch to treatment B for 2 weeks, then A again for 2 weeks, and then B again. For comparison, the following 8 treatment categories were available: acetaminophen; any nonsteroidal anti-inflammatory drug; opioid combination product with codeine; opioid combination product with hydrocodone; opioid combination product with oxycodone; tramadol; complimentary or alternative treatments such as massage, meditation, or physical exercise; and current ongoing therapy (or no therapy). The following 2 types of PGHD were produced during the trial: tracking data and summary data (ie, comparative results).

### Tracking Data

Intervention patients were queried weekly on their adherence to their assigned treatment over the previous 7 days. They were prompted to use the app to provide daily symptom reports on 3 dimensions of pain—pain on average, pain interference with enjoyment of life, and pain interference with daily activities of living. Participants also received daily prompts to report on the following 5 potential side effects of treatment: drowsiness, fatigue, constipation, sleep problems, and cognitive impairment. Patients could enter text as part of their regular reporting. They could view these symptom and side effect reports as simple time line graphs, as seen in Figure 1, anytime during and after their N-of-1 trial. These daily and weekly reports from intervention patients constituted their own tracking data*.* The example graph in Figure 1 shows trends in patient-reported pain and sleep problems.

### Summary Data

**Figure 2:**
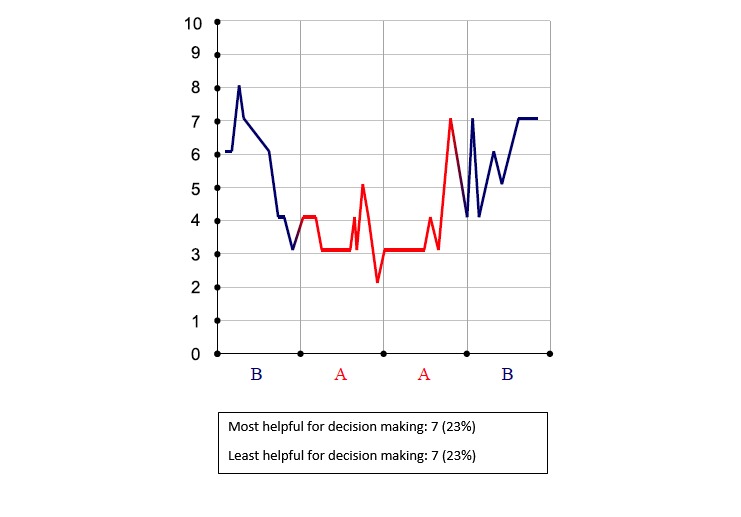
Pain intensity chronology (zero is no pain).

**Figure 3:**
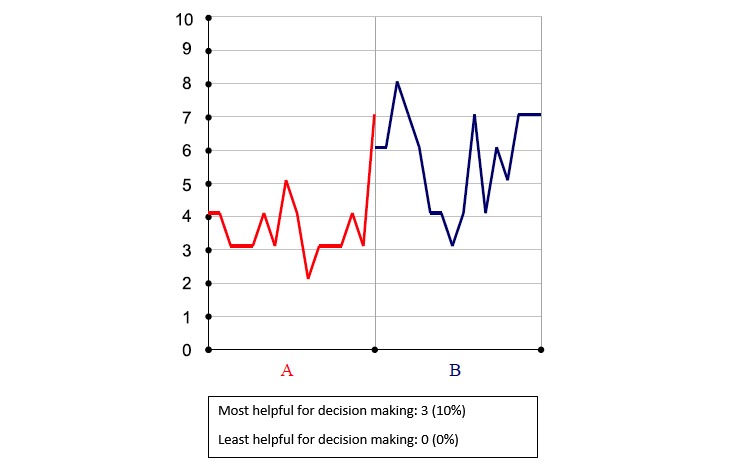
Pain intensity by treatment (zero is no pain).

**Figure 4:**
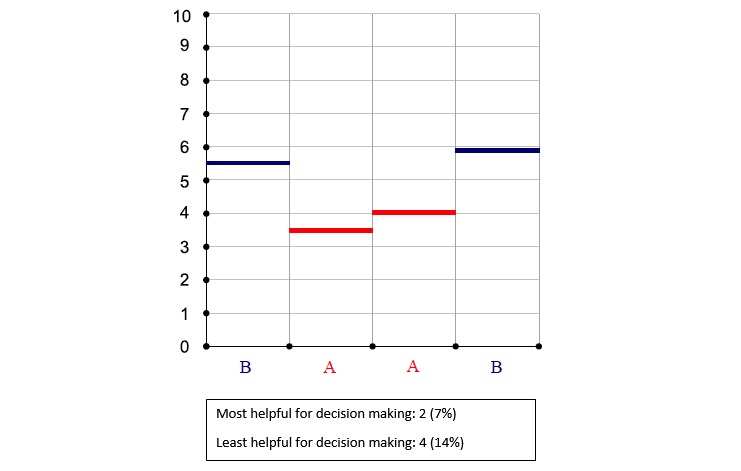
Pain intensity average (zero is no pain).

**Figure 5:**
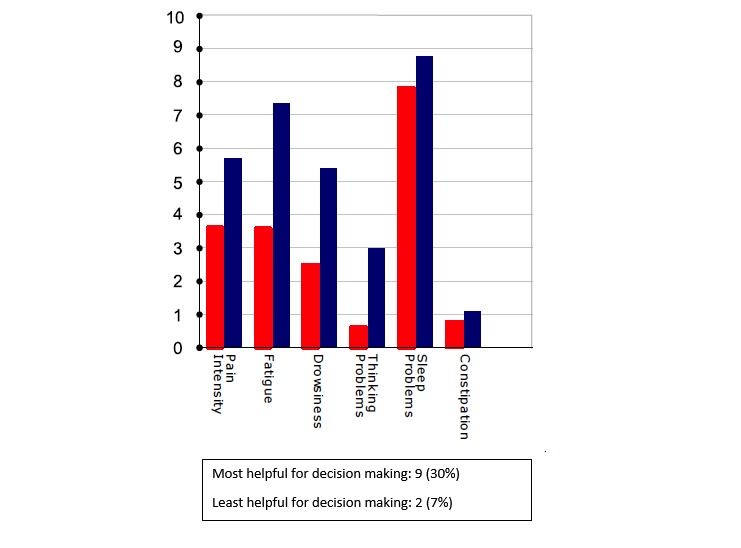
Averaged secondary outcomes (shorter bar is better outcome).

**Figure 6:**
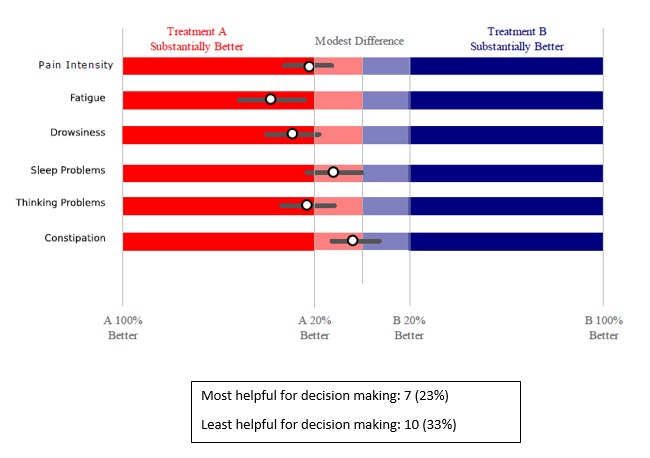
Estimated difference between treatments (bars represent margins of error).

**Figure 7:**
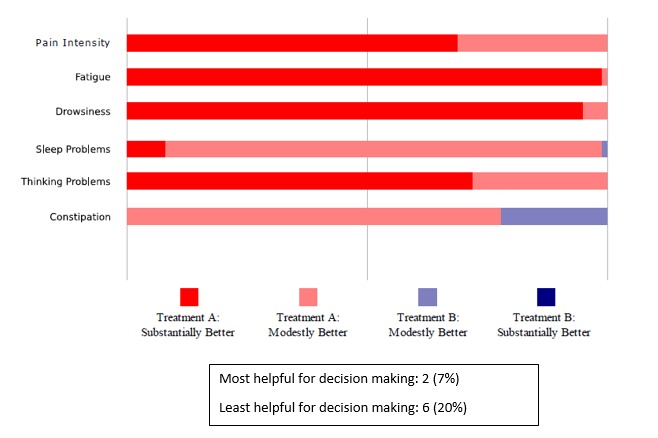
Likelihood that one treatment is better than the other.

Treatment comparison data were made available at the end of a trial. At that time, results comparing the 2 treatments could be viewed by both patient and clinician during a results review visit. Results were presented in 6 different graphical displays; 3 graphs focused on changes in pain intensity over the N-of-1 trial (Figures 2, 3, and 4), 1 bar graph compared Treatments A and B for pain intensity and side effects (Figure 5), and the final 2 graphs provided estimates of changes in the effect and the probability that one treatment was better than another (Figure 6 and 7). These graphs displaying participants’ summary data were the basis on which patients and clinicians evaluated their N-of-1 trial results, as seen in Figure 2.

For this study, a sample of patients was interviewed after the results review visit about their use of the Trialist app and their experiences with their own PGHD including the tracking data compiled from participants’ daily and weekly reports and summary data displayed at the end of an N-of 1 trial. We report on those patient interviews.

### Data Collection and Analysis

The research team designed this qualitative study once the larger study was underway. A patient interview guide was developed. The semistructured interview consisted of questions and open-ended prompts, which were designed to assess the usability and acceptance of the Trialist app, understanding of individualized (N-of-1) study results, preferences for presentation of summary data, and perceptions about PGHD and the process of compiling it.

A consecutive sample of patients from the intervention group was invited to participate in qualitative interviews from February 2016 to September 2016, an 8-month period within the parent study, which lasted from May 2014 to April 2017. Patients were invited at randomization, and those who had completed the N-of-1 trial and were scheduled for a results review visit were interviewed separately from their collaborating clinician. Interviews were completed immediately following the results review visits, which took place an average of 31 days after completing the N-of-1 trial. Because qualitative interviews were designed when the study was already underway, not all potentially eligible patients completed N-of-1 trials and results review visits during the interview period.

Institutional Review Board approval was obtained from University of California, Davis (#496804) and Veterans Affairs, Northern California Health Care System (#13-12-00717) for all aspects of the study, including a modification for these patient interviews that had not been part of the original study design.

Interview data were organized using Dedoose Version 7.1.3 (Los Angeles, CA: SocioCultural Research Consultants, LLC). A descriptive thematic analysis was conducted to identify themes related to patient experiences and perceptions. Repeated reading and analyst triangulation were employed to enhance the quality and validity of themes [12,13]. Sociodemographic characteristics of the interviewed patients were obtained from data collected for the main PREEMPT trial. During their interviews, patients were asked to self-report any background in mathematics, statistics, engineering, science, or health care (yes or no). The research team explored whether there were apparent differences in patient experiences or preferences related to this background.

## Results

### Patient Characteristics

Overall, 215 patients with chronic musculoskeletal pain were randomized into the RCT. Altogether, 107 control group patients received usual care and 108 intervention patients were supplied with the Trialist app; 10 patients in the intervention group did not start a trial, and 3 patients stopped the trial too early to have any results. The remaining 95 patients completed their planned N-of-1 trials. Of these, 80 completed an in-person results review visit, 2 patients used an electronic patient portal for the discussion with the clinician, and 13 patients received N-of-1 trial results by mail.

**Table 1:** Sociodemographic characteristics of Personalized Research for Monitoring Pain Treatment participants who participated in qualitative interviews (N=33).

Characteristic		Participants
Age (years), mean (SD)		55 (10)
**Sex, n (%)**		
	Female	15 (45)
	Male	18 (55)
**Race or ethnicity, n (%)**		
	White, non-Hispanic	22 (66)
	Black, non-Hispanic	3 (9)
	Asian, non-Hispanic	1 (3)
	Hispanic or Latino	5 (15)
	Other	2 (6)
**Highest completed education, n (%)**		
	High school diploma or equivalent (general educational development)	1 (3)
	Some college (associate’s degree or vocational training)	16 (48)
	College degree (bachelor of arts and bachelor of science)	8 (24)
	Graduate degree	8 (24)
Self-reported background in math, statistics, engineering, science or health care, n (%)		19 (58)
Married, n (%)		20 (61)
Employed (full or part time), n (%)		16 (48)

During the qualitative interview period, 46 patients were randomized to the intervention group. Of these, 33 patients were interviewed after their results review visit, 5 potentially eligible intervention patients did not start a trial, 6 did not consent to the interview, and 2 consented but did not complete the interview. The demographics of the 33 interviewed patients are displayed in Table 1.

From our analysis, 3 major themes emerged with subthemes for each. The themes are summarized along with illustrative quotations in Textbox 1.

1. Patients were enthusiastic about *accessing* their PGHD.
2. Patients found value in *sharing* data with their clinicians.
3. Patients engaged energetically in *using* their own data for a wide range of purposes, some apart from those anticipated by investigators.

**Summary of themes and subthemes with illustrative quotations in italics.**

1. Accessing Data
  - Value of tracking data
    - *I think that documenting it was the most helpful.*
    - *The most important factor for me was the diary.*
  - Simplicity of presentation
    - *These are too confusing for me...I want simple.*
    - *It’s like homework...I don’t think so.*
  - Alternatives to data presentation
    - *I would like to see a little person—maybe some more pictures...I mean I understand the bars, but I get excited when I see a little girl riding a bike or somebody going up an [in]cline [to represent changes in pain].*
2. Sharing Data
  - Patient-clinician relationship
    - *We [patient and clinician] had a nice interchange about elaborating on why the results were the way they were. You can only do that sitting side by side together.*
    - *She [the clinician] gets to know how my feet are truly feeling.*
  - Confusion about data analysis
    - *She [the clinician] had no idea what the graphs meant.*
3. Using Data
  - Noticing: increasing self-awareness
    - *I think documenting it was most helpful because then I could see how many days I’d been a space cadet.*
  - Deciding: drawing conclusions and correlations
    - *[The app] helped me draw my own conclusions.*
    - *I guess it just made you pause...think about your day. What you did. How it felt. Those things, on a daily basis, I think it is a useful exercise in pain management.*
    - *Well, I was confused until he [the clinician] explained it...The clinician [was the most influential factor in making a treatment decision].*
  - Acting: self-management
    - *I was also accountable when I wrote notes when I could say I didn’t do this or I didn’t do that or if that increased my fatigue...it made me think about what could be causing some other things and if I needed to address it.*
    - *It let me play around with do I need one [medication dose], do I need to take two, do I need to take ‘em six hours apart, do I need to take ‘em eight hours apart.*
    - *Door number three, that I came up with, worked better...the last half of the trial I was on Plan C [his own plan].*

### Accessing Data

#### Value of Tracking Data

Nearly all interviewed patients expressed enthusiasm about having access to their tracking data. They valued having recorded data as well as its presence in a format to which they could refer. “[T]he most important factor for me was the diary,” said one respondent (female, 50s), whereas another said, “I think that documenting it was the most helpful...” (female, 70s). A patient (male, 40s) who stated he had “a little TBI (traumatic brain injury)” credited the tracking as a memory aide.

#### Simple Presentations of Summary Data

Critically for the N-of-1 context in which treatment comparisons were to be made, a majority of respondents preferred simple presentations of summary data. A variety of graphical presentations of data summarizing trial results were provided to patients and clinicians at the results review visit. As part of the patient interview, respondents were asked which graphs they preferred. Of 33 patients in this subsample, 42% (14/33) did not identify themselves as having a background in mathematics, engineering, science, or the health professions (Table 1), but even those who did (19/33, 58%) expressed preference for simple data presentations of N-of-1 trial results. In choosing which graph was the most helpful to them in making a treatment decision, just over half of patients (16/30, 53%) found either Figure 6 or 7—the two summary graphs that included information about effect size and statistical uncertainty—least helpful for decision making, as seen in Figures 2-7. A footnote to each graph in Figures 2-7 displays the number of interviewees (of 30 who responded to this question) who endorsed that graph as either the most or least helpful in making a treatment decision.

One patient requested that clinicians “spare the patient” the work of complex interpretation of graphs. Another suggested that patients would lose interest in an array of data: “Keep it to the point” (male, 50s). Another interviewee said the following in reference to the more statistically oriented graphs, as seen in Figures 6 and 7: “These are too confusing to me...I want simple” [male, 40s].

Referring to one of the graphs (Figure 6), a respondent said “It’s like homework. I don’t think so” [female, 70s].

#### Alternative App Designs

Many patients proposed a number of innovative suggestions for alternative ways to design the app and showed a range of preferences for data presentation; for example, one respondent (female, 50s) wanted to be able to track more than one pain source, whereas another (female, 50s) suggested creating a link to pain management tips when pain scores were high. Patients suggested changes in the app’s design to increase their own motivation to input their data—one respondent (female, 40s) suggested adding graphics, specifically a bicycle going up a hill, to illustrate effort and progress. Another (male, 60s) suggested varying questions’ order to prevent rote and unthinking responses to repeated queries about pain-related symptoms such as fatigue:

When the questions are repetitive, people tend to know exactly what to hit every single day and it’s done. They shoulda come up with different questions that meant the same, but would make the person read ‘em, instead of knowing what to put, if I’m being honest.

Some respondents wanted compression of data, whereas others sought more detail.

### Sharing Data

#### Value to Patient-Clinician Relationship

Patients were questioned about the format for results of review visits with clinicians and asked if email or phone visits to review the N-of-1 trial results summary data would have been as satisfactory as an in-person visit. Most respondents preferred to meet in person with clinicians to discuss results. One patient with a high level of statistical knowledge stated,

We [patient and clinician] had a nice interchange about elaborating on why the results were the way they were. You can only do that looking at it side-by-side together.

male, 70s

Another, without statistical expertise, explained a preference for face-to-face contact saying, “It’s easier to have a discussion and ask questions if someone’s right in front of you” [female, 50s].

Some patients specifically reported that they engaged their clinicians more effectively when they could back up symptom reporting with summary data. A patient (male, 40s) engaged in an appeal for disability services was enthusiastic about graphed data that would provide “...proof that–for my appeal–to have proof of a graph.” Another (male, 50s) suggested that the tracking data generated in real time added credibility to communication with the clinician: “She [the clinician] gets to know how my feet are truly feeling.”

Another (female, 50s) was able to collect data on the symptom that troubled her the most (constipation) and bring it to the clinician’s attention as they studied trial results together. Others reported improved relationships with their clinicians from engaging in the N-of-1 trials using PGHD.

#### Understanding Data Analysis

A few patients admitted their lack of understanding about data analysis. In the portion of the interview reviewing data analysis for the N-of-1 trial, 2 patients relied on the staff interviewer to clarify N-of-1 data. One (female, 70s) exclaimed, upon viewing graphs not previously viewed with the clinician, “Oh, these are cool...I never read this. ” After the interviewer briefly explained the data, another respondent (male, 50s) said that the data had been “confusing, but once it’s explained...”

Some respondents perceived that their clinicians were also uncertain about data analysis. Several patients reported that their clinicians did not look at the graphical summary data during the results review visit. Some patients reported that their clinicians had limited interest in PGHD, and a few suggested that clinicians, like themselves, might not have understood the data summaries. While reporting that his clinician did not refer to summary graphs, a patient (male, 60s) stated that the clinician had admitted that she did not understand them: She [the clinician] had no idea what the graphs meant.

Several respondents suggested that adding text explanations to summary graphs would have facilitated interpretation, presumably for both patients and clinicians.

### Using the Data

#### Noticing

Overwhelmingly, patients reported noticing what they had not noticed before from daily reporting of their symptoms. One patient said “I think that documenting it was the most helpful...[because] then I could see how many days I’d been a space cadet” [female, 70s].

One respondent (female, 50s) found that the app clarified some specifics about her condition, for example, the role constipation played in her well-being. Patients remarked on their enhanced awareness of symptoms, for example, noting that the trial identified issues with sleep, activity, medication use, and others.

#### Deciding: Drawing Conclusions and Correlations

Most patients described interacting with their tracking data such that it moved them beyond noticing, toward drawing conclusions and correlations from their data. One respondent (male, 50s) said about the app, “It helps you understand the interaction between your sleep and your pain, between your activity and your pain.” Another patient (female, 50s) praised the app’s tracking function, saying it made her “sit and think about it. I guess just make you pause...think about your day. What you did. How it felt. Those things, on a daily basis, I think is a useful exercise in pain management.”

Similarly, another patient (female, 50s) described enhanced self-awareness leading to better pain management:

It definitely helped me pay attention to what was helping alleviate my pain. It felt like I was more mindful.

One patient (male, 60s) said, "It [tracking] just triggered me to start looking at my overall health." Several patients spoke of the conclusions and correlations they drew from their data: "[The app] helped me draw my own conclusions [male, 30s].

Patients had varied reactions to different pieces of their data. One respondent (male, 50s), on being asked what was most influential in coming to a treatment decision, suggested that the trial results were less important “It was more the fact that I was actually entering and writing down.” Another respondent (male, 50s) was also enthusiastic about the graphs of daily symptom reports within the app, stating the following:

Cool thing about the app and these graphs is it helps you understand the interaction between your sleep and your pain, your activity and your pain. Between your pain and your social life and your work life and your home life.

About the collected data, another (female, 40s) said “It validated what I kind of already knew inside.” However, several patients, particularly those who felt more confusion about what graphs meant, reported that the clinician’s input was the main factor in deciding which treatment was better:

Well, I was confused until he [the clinician] explained it...The clinician [was the most influential factor in making a treatment decision].

female, 50s

#### Acting: Self-Management

Most patients used the tracking data to guide self-management. One (male, 40s) reported that he would not have tried an alternative treatment without the app. Another (male, 50s) suggested a higher level of accountability for health behavior from documenting:

I was also accountable when I wrote notes when I would say I didn’t do this or I didn’t do that or if that increased my fatigue...it made me think about what could be causing some other things and if I needed to address it.

Another respondent (male, 50s) stated that symptom report graphs were valuable and reinforced a changed approach,

I [used to] let it [pain] peak too much. Now I’ve gotten it down, and I can really tell by the graph.

Some patients described further steps in self-management. Patients used data to identify triggers they subsequently worked to avoid or data that motivated improved attention to activity or socializing. Patients made alterations in the dose, frequency, and other aspects of treatment regimens. One patient said:

“It [data from the app] let me play around with do I need one [medication dose], do I need to take two, do I need to take ‘em six hours apart, do I need to take ‘em eight hours apart.”

female, 50s

Another (male, 50s) echoed this, having tracked his dose response:

In terms of dosage and the frequency, it allowed me to experiment with how much I was taking and how often I was taking it in terms of dealing with that pain.

A few patients revealed highly independent decisions about their treatment approaches. When asked if he had a sense from summary data that one treatment in the N-of-1 trial was better than another, one patient (male, 70s) answered:

Yeah, mine...I mean, I don’t know if they would accept that for an answer, but door number three, that I came up with, worked better...The last half of the trial, I was on Plan C [his own plan].

Another (male, 50s) said “It [viewing the summary data via the app] helped me decide going forward my next strategy.”

Many patients reported on their own analyses of their data. One respondent (male, 40s) commented on using the app to “not give me the answer but give it [data] to me so I can say ‘ok, mmm...’.”

Another patient (male, 30s) spoke at length about using data independently:

It was good for me to be able to correlate things even before there was some sort of analysis presented to me.

However, this same patient also said of the summary analyses

[E]ven though it’s my own information and I’m putting it into it [the app], having it presented that way [in summary graphs] back to me was helpful.

This patient suggested an increased level of patient use of data:

I think making that app available to–or I mean, could you make that app available to people just to use on their own–I don’t know–to present that monthly analysis of something like that to where they could do their own?...[D]raw my own conclusions about what was going on with my health.

## Discussion

### Principal Findings

As is true in qualitative research, these results are not representative, but rather illustrate issues for discovery and discussion. Our findings from a consecutive sample of the intervention group in a randomized controlled trial examining smartphone-enabled N-of-1 trials for chronic pain (The PREEMPT Study) reinforced previous findings about patient enthusiasm for access to their own PGHD and benefits to N-of-1 trial participation that included increased awareness of symptoms [9,10,14-16].

Participants demonstrated clear preference for simple presentations of summary data. Although few studies have reported on patient preferences for data presentation in the N-of-1 trial context, previous studies examining patient preferences for the presentation of risk-benefit information have similarly found that patients often prefer more simplistic data presentations [17,18]. However, there is a variability in what patients consider “simple” or “understandable.” Respondents in our study were eager to personalize the content and display of their PGHD within the app in the way that made the most sense to them. Tait et al (2012) found that patients who were given risk-benefit information that matched their preferred data visualization format (eg, if their preferred format was a pictograph, the information was presented to them as a pictograph) not only reported greater satisfaction with information but also interpreted it with significantly greater accuracy [19]. As clinicians and health systems work toward shared health decision making and improved chronic condition management, they may find it important to assess patients’ preferred styles of data presentation. In future use of their own PGHD, patients might use their preferred method of tracking symptoms and receiving comparative treatment trial results. With appropriate cautions about overinterpreting data, patients could be provided with presentations of data most useful and motivating to them.

Although our research group was concerned about the tradeoff between accuracy and simplicity of statistical results, no patients expressed similar concerns. We might assume that our respondents did not fully understand statistical uncertainty, but we did not test our patients’ knowledge nor did we teach a primer on statistical interpretation. Future N-of-1 studies might incorporate these additions. Some patients expressed confusion about the meaning of statistical summary data, and almost all expressed a preference for simplicity of results. In the primary study, 23% of N-of-1 trials completed (22/95) did result in significant statistical differences in treatment regimens (ie, a 95% credible interval that excluded the null value) [20]. The research team was concerned that patients would confuse statistical and clinical significance, perceiving greater clinical meaning from lesser statistical difference, especially in trials in which the difference was not significant. Those concerns were borne out during interviews when patients reported that summary graphs with the most information about effect size and statistical uncertainty were the least helpful for decision making. Instead, patients appeared primarily concerned with finding any differences between treatment regimens and in being able to use data to confirm their qualitative perceptions. The growth of patient-centered research approaches may allow for a sophisticated development of consumer friendly data analyses that also meet the bar for understandability and accuracy, in particular, accurately conveying the amount of uncertainty.

We found that although patients used their data autonomously for self-management, they also wanted to partner with their clinicians to review the results and make decisions. A previous study that examined clinicians’ perspectives on N-of-1 trials revealed concerns about the potentially negative impact on the clinician-patient relationship [7]. However, no patients in our study reported such concerns. On the contrary, most felt that discussing trial results with their clinicians was valuable and important. The optimum way for clinicians and patients to share N-of-1 trial data remains to be determined; our study only hints at patients’ perceptions from sharing their data as well as at a caution that clinicians too will need education about interpreting these data.

Our study suggested that patients evaluated the tracking data and summary data distinctively. Patients were enthusiastic about the tracking data and somewhat less so about summaries to which they had only brief exposure. The main study was designed to collect daily symptom reports for the primary purpose of comparing the effectiveness of two alternative treatment regimens. However, qualitative analysis reported here reveals that patients did not see the comparison function as the only, or possibly even the main, value of their PGHD. Instead, they appreciated the ability to track their symptoms over time with perhaps greater motivation because an eventual comparison was in store. This may be a case of the process having as much or more value than the outcome alone. These findings are consistent with those of another qualitative study, which found that patients were primarily motivated to participate in N-of-1 trials to increase accountability for self-management and promote behavior change rather than to compare treatments [21].

Importantly, our study focused on chronic pain and relied on patient self-report for the primary outcome. Because patients were experiencing and reporting any changes in pain throughout the trial, the final comparative effectiveness result may have been less meaningful. Future work might explore whether patients report more benefit from comparative effectiveness results in situations where the main outcome is important to patients, but not as easily perceived by them (ie, cholesterol and A1C).

Some patients expressed confusion about the summary data’s meaning, and almost all expressed preference for simple presentations of trial results. This suggests that to engage patients deeply in their own N-of-1 trials could mean designing analysis strategies more transparent to patients, in addition to being easily understood. Could patients choose analyses they want with simple explanations of tradeoffs between comprehensiveness and comprehensibility? One might program different interpretations for different types of patients (eg, one for the math phobic and another for the data enthusiast). In addition, what ways are there to bring data analyses to patients? Are clinical visits the optimum setting, or are there other forums in which comparative data analysis could be viewed and discussed? Notably, participants in our study were a fairly educated group with about half reporting a college or graduate degree and 58% (19/33) reporting a background in mathematics, statistics, engineering, science, or health care. Future work might explore whether enthusiasm for and engagement with PGHD is similarly high in populations with less education and more limited prior experience with mathematics, statistics, engineering, science, or health care fields.

Although not the focus of these interviews, participants revealed a well known, but sometimes unacknowledged, aspect of clinical practice that patients modify and enact their own treatment regimens. Our interviews showed a wide range of therapeutic actions by patients, at times independently of their clinicians and N-of-1 protocols. Like clinicians, N-of-1 trial researchers should be aware of this and may consider adaptive designs that would enable patients and clinicians to partner in modifying initial trial protocols.

### Conclusion

Notes and numeric data tracked and analyzed by the Trialist app in N-of-1 trials make possible new conclusions and decisions about both collaborative treatment and self-management. Structured records in the app may be shared with clinicians who weigh in with their professional input, a conversation that might not take place without the app’s facilitation. Patients in our study valued sharing PGHD with clinicians, even as they also made their autonomous uses of data. Future N-of-1 studies might consider individualized, adaptable protocols and emphasize clarity of data presentation to optimize shared decision making.

## Abbreviations

PGHD: patient-generated health data
PREEMPT: Personalized Research for Monitoring Pain Treatment
RCT: randomized controlled trial
